# Prolonged Inhibition of *Streptococcus mutans* Growth and Biofilm Formation by Sustained Release of Chlorhexidine from Varnish Coated Dental Abutments: An *in Vitro* Study

**DOI:** 10.1155/2022/7246155

**Published:** 2022-10-14

**Authors:** Mark Feldman, Walid Shaaban Moustafa Elsayed, Michael Friedman, Irith Gati, Doron Steinberg, Hesham Marei

**Affiliations:** ^1^Biofilm Research Laboratory, Institute of Dental Sciences, Faculty of Dental Medicine, The Hebrew University of Jerusalem, Jerusalem, Israel; ^2^Department of Basic Medical and Dental Sciences, College of Dentistry, Gulf Medical University, Ajman, UAE; ^3^The Institute for Drug Research, School of Pharmacy, The Hebrew University of Jerusalem, Jerusalem, Israel; ^4^Department of Diagnostic and Surgical Dental Sciences, College of Dentistry, Gulf Medical University, Ajman, UAE

## Abstract

**Background:**

It has been confirmed that bacterial biofilm covering dental implants is the main microbial source causing preimplant infectious and inflammatory diseases. The purpose of this study was to evaluate the antibacterial/antibiofilm effect of chlorhexidine, incorporated into a sustained-release varnish of chlorhexidine (SRV-CHX) coating, on dental abutments.

**Materials and Methods:**

Three kinds of dental abutments were used: a high-performance semi-crystalline engineering thermoplastic polyetheretherketone (PEAK) healing abutment, a titanium healing abutment, and a titanium permanent abutment. These abutments were coated with SRV-CHX or SRV-placebo and exposed daily to fresh cultures of *Streptococcus mutans*. The effect of SRV-CHX on *S. mutans* growth on agar plates was studied by measuring the zone of inhibition (ZOI) around each tested abutment every day for a period of 36 days. Biofilm formation on the SRV-CHX/placebo-coated abutments was detected using confocal laser scanning microscopy (CLSM) and high-resolution scanning electron microscopy (HR-SEM), energy dispersive X-ray analysis (EDX), and monitored by crystal violet (CV) staining.

**Results:**

SRV-CHX-coated abutments 2 and 3 were able to inhibit *S. mutans* growth for 34 days, while abutment 1 inhibited growth for 32 days. Abutment-associated biofilm formation was notably inhibited by SRV-CHX coating after 13 days of incubation with *S. mutans*. Finally, the biofilm formed around SRV-CHX-coated abutments was completely inhibited up to 12 days of abutment exposure to *S. mutans*.

**Conclusion:**

Coating of dental abutments with SRV-CHX demonstrated long-term effective inhibition of *S. mutans* growth and biofilm formation on the abutment surface.

## 1. Introduction

Peri-implantitis and peri-implant mucositis are the most common preimplant infectious inflammatory diseases [1, 2]. These inflammatory diseases may lead to massive bone resorption, compromising the success and survival of the dental implants [3]. It has been confirmed that bacterial biofilm covering dental implants is the main microbial source causing preimplant infectious and inflammatory diseases [4, 5]. When the biofilm-containing bacteria reach the far tip of the implant, this will result in inflammation and destruction of the bone supporting the dental implants [1–3, 6].

Biofilms are thick and complex bacterial microcommunities that grow and migrate on the root of natural teeth or the metallic surface of implants [5, 7, 8]. The first step of bacterial biofilm formation is the adsorption of salivary proteins to the implant surface to form a thin film known as acquired pellicles. After that, bacteria start to attach to the acquired pellicles to initialize colonization. The attached bacteria excrete an extracellular polymeric matrix, helping other bacteria to colonize and providing nutrients to the growing biofilm [7–9].

Due to advances in molecular biology, the microbiota related to periodontal biofilm has been well identified. *Streptococcus mutans* (*S. mutans*), besides other anaerobes such as *Fusobacterium* and *Actinobacteria*, has been identified to be the main microorganism during the early stages of biofilm formation [10]. In a recent study conducted by Laosuwan et al. [6] comparing the *S. mutans* bacterial biofilm formation and migration on a tooth root and titanium mini screws, found that the distribution of the dome‐shaped biofilm was similar at both surfaces after 48 hours. However, the study found that there is a significant difference in the migration rate as the biofilm migrates faster on the mini-screw surface compared with those on the tooth root at 48 hours [6]. Another study investigating the important role of *S. mutans* during biofilm formation found that the removal of *S. mutans* from the microorganism ecosystem leads to a large reduction in biofilm volume and slows the biofilm development in the oral cavity [10]. *S. mutans* has been studied and implicated in the pathogenicity of dental implantitis, probably as the early colonizer on the surface, initiating the formation of the anaerobic type of biofilm. Studies evaluating the efficacy of different methods to reduce peri-implantitis have been using this bacterium [11–13].

Inhibiting or removing microbial biofilm are the main effective approaches to prevent peri-implantitis [5]. Chlorohexidine is considered the gold standard antimicrobial agent for controlling biofilm. Local sustained-release delivery systems lead to longer contact times of antimicrobial agents with the implant surface, increasing the effectiveness of the antimicrobial agents [14–17]. Several studies have demonstrated the effectiveness of sustained-release varnish containing chlorhexidine to inhibit dental plaque *S. mutans* [17–19].

In one stage of implant surgery, healing abutments are connected to the implants and stay for an average of 3 months before the fabrication of the final prosthesis. A 3-month microbial analysis of the dental plaque revealed a predominant population of cocci and spirochetes around different types of implant abutment, modulated by the surface proprieties of implant abutment material [20]. Furthermore, there is an implant-abutment junction (IAJ) with almost a 40–60 *μ*m joint/gap between the implant and abutment [21]. Several studies have reported bacterial penetration across the implant abutment interface, which increases the risk of biofilm formation [21–24].

Our study aims to evaluate the antibacterial/antibiofilm effect of coating various dental abutments with chlorhexidine incorporated into a sustained-release varnish of chlorhexidine (SRV-CHX).

## 2. Materials and Methods

This study was approved by the Institutional Review Board (IRB), Gulf Medical University, with reference number “Ref. No. IRB/COD/FAC/90/Jan-2021.”

### 2.1. Abutments

**Figure 1:**
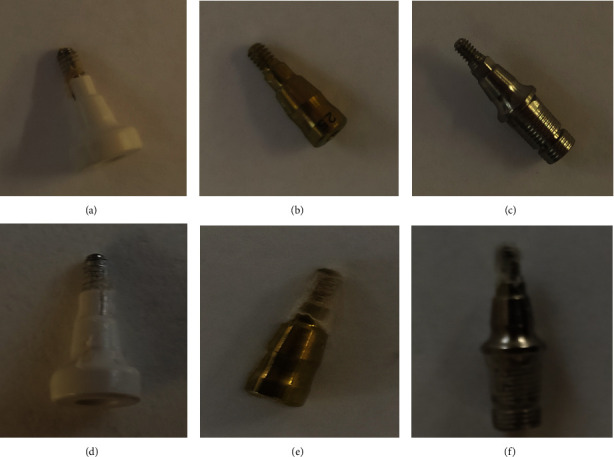
Dental abutments before and after coating with SRV-CHX. Upper panel: three kinds of sterile dental abutments: GM PEAK healing abutment 5.5 × 2.5 mm [1] (a), GM titanium healing abutment 3.3 × 2.5 mm [2] (b), and GM titanium permanent abutment 4.5 × 6 × 2.5 mm [3] (c) were used for this study. Lower panel: the lower screw part of abutments 1, 2, and 3 were coated with the SRV-CHX ((d)–(f), respectively), and allowed to dry aseptically at room temperature.

Three kinds of sterile dental abutments were used: GM PEAK healing abutment 5.5 × 2.5 mm [1] (Figure 1(a)), GM Titanium healing abutment 3.3 × 2.5 mm [2] (Figure 1(b)), and GM Titanium permanent abutment 4.5 × 6 × 2.5 mm [3] (Figure 1(c)) (Neodent, Andover, MA).

### 2.2. Sustained Release Varnish (SRV) Preparation

The SRV-CHX was formulated similarly to that described by Beyth et al. [19]. Briefly, ethylcellulose and polyethylene glycol 400 (PEG 400) were used as the polymeric matrix, and CHX was used as the active antibacterial agent. Ethanol was used as the solvent. The resulting varnish contained 2% (W/V) CHX. The placebo varnish (placebo-SRV) was prepared identically to the experimental SRV, omitting the CHX from the formulation. The wet formulas are presented in Table 1.

### 2.3. Weight of Coating on Abutments

Table 2 represents the dry weight of the SRV coating on different abutments, measured as the difference in weight in mg of the abutment before and after coating.

### 2.4. Bacterial Strain

The bacterial strain used was *Streptococcus mutans* (UA159) (ATCC 700610), which is a model for strongly adhering bacteria to various oral surfaces. Bacteria grown overnight at 37°C, aerobically, at 5% CO_2_, in brain heart infusion (BHI) (Neogen, Lansing, M) were used for the experiments.

### 2.5. Abutments Coating with SRV

The lower screw part of abutments 1, 2, and 3 was coated with the SRV-CHX (Figures 1(d)–1(f), respectively), and allowed to dry aseptically at room temperature. The amount of SRV-CHX was calculated by weighing each abutment before and after coating with the SRV-CHX. In this study, we compared SRV-placebo (coating without CHX) with SRV-CHX. Positive controls were uncoated abutments incubated in brain heart infusion (BHI) supplemented with 1% sucrose containing *S. mutans* at an initial OD_595_ of 0.02. Negative controls were abutments incubated in a BHI medium without bacteria.

### 2.6. Kinetics Experiments

Abutments 1, 2, and 3 coated with SRV-CHX (1-SRV-CHX, 2-SRV-CHX, and 3-SRV-CHX) and SRV-placebo were placed on agar plates pre-seeded with 50 microliters *S. mutans* (0.26 × 107 CFU/mL) at OD595 = 0.02 [25]. The plates were incubated at 37°C aerobically at 5% CO2. The zone of inhibition (ZOI; cm^2^) around the placed abutments was measured daily, and the abutments were removed aseptically and placed on new *S. mutans* pre-seeded plates for further incubations. Three separate experiments were performed. The data are presented as the mean ± SD.

### 2.7. Biofilm Model

SRV-CHX and SRV-placebo-coated abutments were placed in the wells of 24-well plates and inoculated with 2 ml of *S. mutans* at an OD_595_ = 0.02, supplemented with a 1% sucrose final concentration, and SRV-placebo was used as a negative control. Plates were incubated at 37°C aerobically at 5% CO_2_. Biofilm formation around the placed abutments was measured daily using crystal violet (CV) staining (see below), and the abutments were daily transferred to new wells containing fresh *S. mutans* cultures for another 24 hours of incubation. Abutment-associated biofilm formation was monitored using CLSM and HR-SEM after 13 days of exposure to *S. mutans*.

### 2.8. Crystal Violet Staining

To analyze the biofilms formed around the abutments, the wells of the 24-well plates were washed with PBS and incubated with a solution of 0.02% crystal violet (CV) for 45 min, and then washed twice with Double Distal Water (DDW) in order to remove unbound dye. After adding 30% acetic acid into each well, the plate was shaken for 10 min to extract the dye from the biofilms, and the dye was quantified by measuring the absorbance at 595 nm using an Infinite M200 PRO plate reader (Tecan Group Ltd., Männedorf, Switzerland) [26]. Biofilm formation around SRV-CHX-coated abutments was presented as a percentage of biofilm formation around SRV-placebo-coated abutments (100%). The values were determined by three independent experiments, and the data are presented as a mean ± SD.

### 2.9. Confocal Laser Scanning Microscopy (CLSM)

After 13 days of exposing the abutments to *S. mutans*, the formed biofilms were washed twice using PBS. Next, the samples were stained with 2 *µ*M Syto 9 (Invitrogen, Carlsbad, CA) and left for 30 minutes in the dark, then washed again, after which the samples were studied under confocal laser scanning microscopy (CLSM) (Nikon Inc., Melville, NY, USA) with a 488 nm green laser excitation and collecting the data using the 535 nm filter. [14]. At least three random fields were captured for each sample. Three independent experiments were performed, and one set of representative data is shown. The amount of the viable *S. mutans* cells in each sample was analyzed as color-appropriated fluorescence intensity, using Image J v3.91 software (NIH, Bethesda, MD, USA) (https://rsb.info.nih.gov/ij). The total biomass of cells in biofilm formed on SRV-CHX-coated abutments was calculated as a percentage of the total biomass of cells in biofilm formed on SRV-placebo-coated abutments. The data are presented as a mean ± SD.

### 2.10. High Resolution-Scanning Electron Microscopy

The abutment-associated biofilm that formed after 13 days of abutment transfer was washed with PBS and then washed twice using DDW. Then, all samples were fixed using 4% formaldehyde for 20 min and then washed with DDW. To ensure high-quality images, the samples were ultra-thin sputter-coated with iridium prior to observation. The samples were visualized using a high-resolution scanning electron microscope (HR-SEM) (Sirion XL30 SFEG) (FEI, Eindhoven, The Netherlands) at 100x and 5000x magnification. At least four random fields were observed and analyzed. Three independent experiments were performed [27].

### 2.11. Statistical Analysis

The means of independent experiments were calculated. Statistical analysis was performed using the Student's *t*-test, with a *P* value <0.05 considered as statistically significant.

## 3. Results

### 3.1. Prolonged Inhibition of Bacterial Growth by SRV-CHX-Coated Abutments

**Figure 2:**
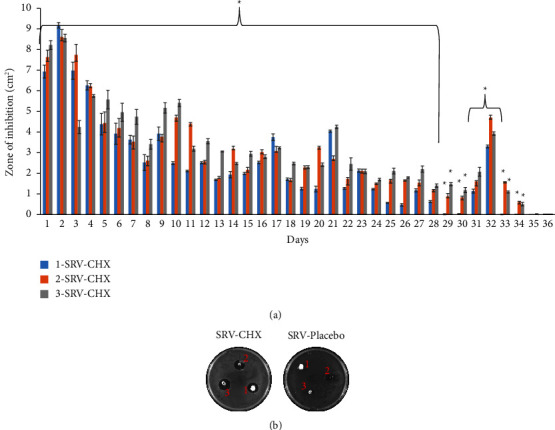
Kinetics of bacterial growth inhibition. Abutments 1, 2, and 3 coated with SRV-CHX (1-SRV-CHX, 2-SRV-CHX, and 3-SRV-CHX) and SRV-placebo were placed on agar plates pre-seeded with 50 microliters *S. mutans* at OD_595_ = 0.02. The plates were incubated at 37°C aerobically with 5% CO_2_. The zone of inhibition (ZOI; cm^2^) around the placed abutments was measured daily, and the abutments were removed aseptically and placed on new *S. mutans* pre-seeded plates for further incubations. (a) Quantitative analysis of bacterial growth inhibition by SRV-CHX-coated abutments [1–3]. ^∗^Significantly higher than the value for SRV-placebo (*P* < 0.05). (b) Images of inoculated agar plates after 21 days of incubation with an inserted 1-, 2-, or 3-SRV-CHX/placebo-coated abutments.

In order to test the long-term inhibitory effect of SRV-CHX-coated abutments on bacterial growth, we performed kinetic experiments. This assay was conducted on *S. mutans*-coated agar plates. The kinetics experiments showed that all tested abutments coated with SRV-CHX delayed bacterial growth in a prolonged manner (Figure 2(a)). Sample 1-SRV-CHX was able to inhibit *S. mutans* growth for up to 32 days, while samples 2-SRV-CHX and 3-SRV-CHX prevented bacterial growth for up to 34 days (Figure 2(a)). Interestingly, all SRV-CHX-coated abutments exhibited wave-like pattern kinetics of growth (Figure 2(a)), which could be attributed to the nonlinear gradual release of CHX.  Figure 2(b) demonstrated a prolonged inhibitory effect of all tested SRV-CHX-coated abutments on bacterial growth after 21 days. SRV-Placebo did not affect bacterial growth.

### 3.2. Inhibition of Abutment-Associated Biofilm Formation by SRV-CHX-Coated Abutments

In order to test the effect of SRV-CHX-coated abutments on abutment-associated biofilm formation, we performed CLSM and HR-SEM assays.

### 3.3. CLSM Assay

**Figure 3:**
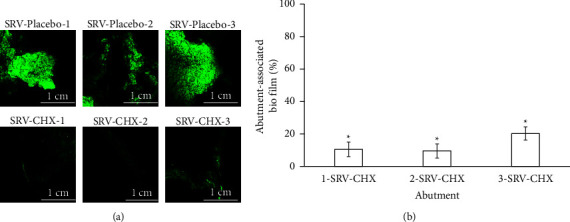
CLSM images of abutment-associated biofilm. *S. mutans* were incubated with sucrose on the SRV-placebo/CHX-coated abutments in order to form biofilms. Cells in biofilms formed on the abutments were examined after 13 days of exposing the abutments to *S. mutans* using a CLSM after staining with SYTO 9 (green fluorescence). (a) Upper panel: SRV-placebo-coated abutments, 1, 2, and 3; lower panel: SRV-CHX-coated abutments, 1, 2, and 3. Magnification ×40. (b) Quantitative analysis of biofilm formation on SRV-CHX-coated abutments. ^∗^Significantly lower than the SRV-placebo (*P* < 0.05).

The total biofilm of viable S. *mutans* cells on the SRV-coated abutments was measured by the fluorescent signal intensity recorded from the surface of the abutments. A low signal was observed in images of SRV-CHX-coated abutments (Figure 3(a), lower panel); while a strong signal intensity of the attached biofilm was demonstrated in SRV-placebo coated abutments images (Figure 3(a), upper panel).

Figure 3(b) shows a quantitative analysis of the images. The amount of viable *S. mutans* cells in biofilm was reduced on all tested abutments coated with SRV-CHX as compared to SRV-placebo-coated abutments. Biofilm formation of *S. mutans* was inhibited by more than 90% on 1-SRV-CHX samples and 2-SRV-CHX samples, and by 80% on 3-SRV-CHX as compared to SRV-placebo (100%).

### 3.4. HR-SEM Assay

**Figure 4:**
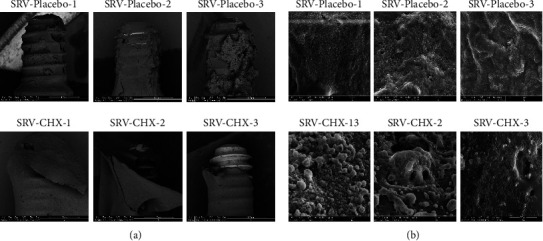
HR-SEM images of abutment-associated biofilm. The abutment-associated biofilms formed after 13 days of abutment exposure to *S. mutans* were visualized using HR-SEM. (a) Upper panel: SRV-placebo-coated abutments, 1, 2, and 3; lower panel: SRV-CHX-coated abutments, 1, 2, and 3. Magnification ×100. (b) Upper panel: SRV-placebo-coated abutments, 1, 2, and 3; lower panel: SRV-CHX-coated abutments, 1, 2, 3. Magnification ×5000.

HR-SEM examination of SRV-placebo-coated abutments showed strong colonization and biofilm formation of *S. mutans* on their surfaces (Figures 4(a) and 4(b), upper panels). In contrast, all abutments coated with the SRV-CHX demonstrated a notable reduction of *S. mutans* colonization (Figures 4(a) and 4(b), lower panels). These results were based only on observation. No detectable biofilm formation is observed on samples 1-SRV-CHX and 2-SRV-CHX (Figure 4(b), lower panel) as there are almost no visible bacteria compared to SRV-Placebo, while an obvious decrease in *S. mutans* biomass is seen on sample 3-SRV-CHX (Figure 4(b), lower panel) as compared to 3-SRV-placebo (Figure 4(b), upper panel). In addition, numerous large aggregates are visible on the surface of 1-SRV-CHX and 2-SRV-CHX (Figure 4(b), lower panel), while few small aggregates appear on the surface of 3-SRV-CHX (Figure 4(b), lower panel).

### 3.5. Inhibition of Well-Associated Biofilm Formation by SRV-CHX-Coated Abutments

**Figure 5:**
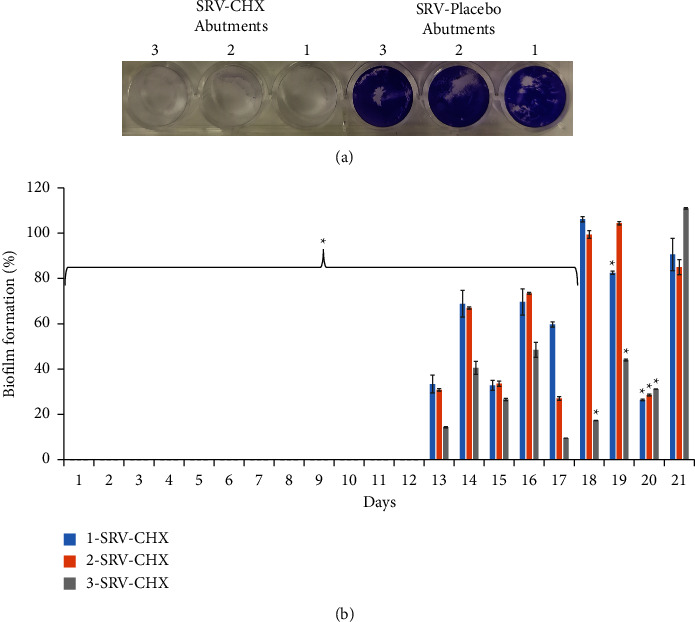
Biofilm formation around SRV-CHX-coated abutments. (a) Biofilm formation after 12 days of abutment passages. SRV-placebo and SRV-CHX-coated abutments 1, 2, and 3; (b) quantitative analysis of biofilm formation around SRV-CHX-coated abutments. The data are presented as a percentage and compared to the SRV-placebo (100%). ^*∗*^Significantly lower than the value for SRV-placebo (*P* < 0.05).

In order to test the effect of SRV-CHX-coated abutments on well-associated biofilm formation, we performed a CV assay. After each day of biofilm formation in the wells with the presence of SRV-placebo/CHX-coated abutments, the biofilms that formed around the abutments were detected by CV staining. Biofilm formation around all tested SRV-CHX-coated abutments was totally inhibited for 12 days as compared to SRV-placebo-coated abutments (100%) (Figures 5(a) and 5(b)). Starting from day 13, the inhibitory potential differs between SRV-CHX-coated abutments. 3-SRV-CHX was the most effective in preventing well-associated biofilm formation by more than 50% up to 20 days as compared to SRV-placebo-coated abutments (Figure 5(b)). Similarly, as detected in the bacterial growth study, wave-like pattern kinetics of biofilm inhibition was observed concerning all tested SRV-CHX-coated abutments (Figure 5(b)).

## 4. Discussion

Placement of dental implants is now a daily routine in dental practice as a reliable treatment for replacing missing teeth. It was found that 5.7% of USA patients have received dental implants in 2016 with five million implants every year [28]. Globally, the dental implant market is expected to reach around $4.5 billion a year by 2022 [28]. Despite the advances in the field of dental implants, the survival of dental implants depends on many factors. Bacterial biofilm, which is the main cause of peri-implant mucositis and peri-implantitis, is the most commonly blamed reason for the failure of the dental implant [1, 2]. Inhibiting the early formation of dental biofilm is very crucial to preventing preimplant diseases. Our study takes advantage of the sustained-release technology as a coating on dental implants.

Our study used different modalities and techniques such as SEM, CLSM, and CV staining demonstrating inhibition of biofilm formation around all tested SRV-CHX-coated abutments. The amount of viable *S. mutans* cells in biofilm was reduced by 80% to 100% on all tested coated with SRV-CHX abutments. These results were also confirmed by the HR-SEM examination, which demonstrated a notable reduction of *S. mutans* colonization of SRV-CHX-coated abutments.

It was found that the microbial colonization of dental biofilm follows the same pattern on dental implants and the teeth [5]. *Streptococci* bacteria are considered the initial colonizers as they have the ability to bind to the tooth surface [29]. *S. mutans,* among other bacteria such as *Actinomyces* species and *S. sobrinus*, is playing an important role during the early stages of colonization and acts as a bridge to the more virulent periodontopathogenic bacteria such as *Porphyromonas gingivalis* (PG), *Tannerella forsythia* (TF), and *Treponema denticola* (TD) to attach and colonize in the mature biofilm [5, 29, 30]. Unlike natural teeth, dental implants lack indigenous places for bacterial biofilm, giving early colonizer bacteria such as *S. mutans* an important role for initiating the biofilm on the implant surface.

Our study demonstrates the ability of the SRV-CHX coating to minimize the number of viable *S. mutans* and prevent its surface colonization. The decreasing number of viable *S. mutans* and inhibiting its colonization on the implant surface may lead to preventing the formation of dental biofilm in clinical scenarios. A study conducted by Koo et al. indicated that the removal of *S. mutans* from the microorganism ecosystem dramatically affects biofilm formation in the oral cavity, leading to a large reduction in biofilm volume and slowing the biofilm's development [10]. Another study indicated that modification of the surface modalities of implants after three different peri-implantitis treatments inhibits the growth of *S. mutans* on the surfaces of implant, resulting in less biofilm formation [29]. Implants are to be placed in the mouth for a long period of time. The initial period of implantation is of great impact for the success of the clinical procedure. Preventing initial biofilm formation during tissue recovery is important for the success rate of any dental or other implant in the body.

These findings support our hypothesis on the important role of *S. mutans* in biofilm formation and the possibility of targeting its colonization as an effective approach for controlling biofilm formation and preventing peri-implant diseases.

Although the microbial colonization of dental biofilm follows the same pattern on dental implants and the teeth, the nature of bacterial biofilm is affected by the surface properties, surface roughness, surface topography, and surface stiffness of implants [31]. It was found that the greater the surface roughness, the higher is the rate of the biofilm formation around the implant [31]. This might explain the difference between the three types of implants, SRV-CHX (1-SRV-CHX, 2-SRV-CHX, and 3-SRV-CHX), in response to the inhibitory effect of biofilm formation and *S. mutans* viability and colonization. In our study, we found that the GM Titanium permanent abutment coated with SRV-CHX was responding in a stronger way than the other coated abutments. This may be due to its surface proprieties affecting the release of the CHX from the SRV. These results are in agreement with other results conducted by other researcher [32], highlighting the importance and influence of the surface roughness and surface free energy in biofilm formation.

Sustained release delivery systems offer many unique pharmacological and clinical characteristics. A sustained-release delivery system allows local delivery of the antimicrobial agent to the targeted site [15, 29]. Additionally, it also minimizes the potential adverse systemic effects. Also, SRV is allowing the antimicrobial drug to be released over a long period of time in a controlled manner. This slow-release advantage increases direct contact time between the drug and implant surface, improving the efficacy of the antimicrobial agents [15, 29].

Chlorohexidine is considered a broad-spectrum antimicrobial agent for controlling oral or nonoral bacterial biofilm [33, 34]. It is commercially used in many dental (mouthwashes, toothpastes, periodontal pocket inserts) and nondental medications (dermatology, vaginal infections). The SRV basic formulation has been used in several clinical studies with no adverse effects [35]. Moreover, the SRV-CHX has also been used in several animal and human studies with no adverse effect [19, 33, 36].

In our study, we have proved the pharmacological ability of our SRV to keep the local concentration of the CHX in appropriate concentration for a long time, as it was proved by EDX pattern showing the presence of CHX even after 13 days, and its efficacy to prevent biofilm formation over a long period. Interestingly, after the period of total inhibition of biofilm formation, we observed increases and decreases in biofilm formation around all tested SRV-CHX-coated abutments. This phenomenon could be explained by the specific surface properties of each type of abutment, which give a wave-like release pattern that retains the release of the CHX from the SRV. The graduate release of CHX was efficient up to 21 days; after this period, no significant effect on biofilm formation was detected (data not shown), indicating that most of the CHX has been released from the SRV.

In summary, the sustained-release delivery technologies have been tested and used in many dental applications. In this study, we tested the *in vitro* feasibility of those pharmaceutical systems in dental implants as well. Our results showed the ability of the SRV-CHX-coated abutment to inhibit the formation of dental biofilm, decrease the number of *S. mutans,* and inhibit its colonization on three different types of dental implant abutments. These results will lead to extended *in vitro* and then *in vivo* studies on SRV application in dental implants.

## Figures and Tables

**Table 1:** The wet formula of SRV-CHX and SRV-placebo.

	SRV-CHX	SRV-placebo
Chlorhexidine diacetate	0.59 mg	
Ethylcellulose	2.35 mg	3.03 mg
Polyethylene glycol 400	0.235 mg	0.3 mg
Ethanol	29.5 ml	29.5 ml

**Table 2:** The dry weight of coating on abutments, mg.

	Abutments		
	1	2	3
SRV-placebo	3.1	3.2	3.2
SRV-CHX	3.2	3.3	3.5

## Data Availability

The data used to support the findings of this study are openly available in NCBI at https://www.ncbi.nlm.nih.gov/.

## Additional Points

What is known about the topic: (i) Bacterial biofilm is the main microbial source causing preimplant infectious and inflammatory diseases. (ii) *Streptococcus mutans* (*S. mutans*), besides other anaerobes, has been identified to be the main microorganism during the early stages of biofilm formation. (iii) Inhibiting or removing microbial biofilm are the main effective approaches to prevent peri-implantitis. What the submitted study adds: (i) The ability of the sustained-release varnish of chlorhexidine (SRV-CHX) coating to minimize the number of viable *S. mutans* and prevent its surface colonization on dental abutments.
